# Major Breeding Plumage Color Differences of Male Ruffs (*Philomachus pugnax*) Are Not Associated With Coding Sequence Variation in the *MC1R* Gene

**DOI:** 10.1093/jhered/esu079

**Published:** 2014-12-22

**Authors:** Lindsay L. Farrell, Clemens Küpper, Terry Burke, David B. Lank

**Affiliations:** From the Department of Animal and Plant Sciences, University of Sheffield, Sheffield S10 2TN, UK (Farrell, Küpper, and Burke); and the Department of Biological Sciences, Simon Fraser University, Burnaby, British Columbia V5A 1S6, Canada (Farrell and Lank). Lindsay L. Farrell is now at Roslin Institute, University of Edinburgh, Easter Bush Campus, Midlothian EH25 9RG, UK.

**Keywords:** *MC1R*, *melanism*, *pigmentation*, *plumage variation*, *sequence variation*

## Abstract

Sequence variation in the *melanocortin-1 receptor* (*MC1R*) gene explains color morph variation in several species of birds and mammals. Ruffs (*Philomachus pugnax*) exhibit major dark/light color differences in melanin-based male breeding plumage which is closely associated with alternative reproductive behavior. A previous study identified a microsatellite marker (*Ppu020*) near the *MC1R* locus associated with the presence/absence of ornamental plumage. We investigated whether coding sequence variation in the *MC1R* gene explains major dark/light plumage color variation and/or the presence/absence of ornamental plumage in ruffs. Among 821bp of the *MC1R* coding region from 44 male ruffs we found 3 single nucleotide polymorphisms, representing 1 nonsynonymous and 2 synonymous amino acid substitutions. None were associated with major dark/light color differences or the presence/absence of ornamental plumage. At all amino acid sites known to be functionally important in other avian species with dark/light plumage color variation, ruffs were either monomorphic or the shared polymorphism did not coincide with color morph. Neither ornamental plumage color differences nor the presence/absence of ornamental plumage in ruffs are likely to be caused entirely by amino acid variation within the coding regions of the *MC1R* locus. Regulatory elements and structural variation at other loci may be involved in melanin expression and contribute to the extreme plumage polymorphism observed in this species.

Birds display a wide range of variation in plumage coloration and pattern that has long fascinated biologists because of its importance in sexual selection, speciation, and adaptation (Roulin 2004). The ruff (*Philomachus pugnax*) is a lekking sandpiper which exhibits major dark/light color differences in melanin-based male breeding plumage that is closely associated with a genetic polymorphism for alternative male mating behavior (Hogan-Warburg 1966; Van Rhijn 1973; Lank et al. 1995). Three genetic male morphs persist in ruff populations: 1) dark-plumed territorial “Independents”; 2) light-plumed non-territorial “Satellites”; and 3) small female-like males called “Faeders” that lack ornamental plumage (Jukema and Piersma 2006; Lank et al. 2013). The extensive individual variation in melanin-based coloration of ornamental neck ruffs and head tufts of male ruffs has been well described (Hogan-Warburg 1966; Höglund and Lundberg 1989; Ekblom et al. 2012; Van Rhijn et al. 2014) and a wide range of plumage colors and patterns exists within both independent and satellite morph types. Independents range from black, dark rust, light rust to ivory, with occasional patches of white, but always contain predominantly dark rust or black feathers in ruff, head tufts, or both. In contrast, satellites are predominantly white in ornamental plumage color, but range from white, ivory to straw yellow, with secondary ruff colors of light to medium rust, but lack solid black feathers in the ruff or head tufts (Figure 1). This hypervariability has been attributed to diversifying selection for individual identity signalling (Lank and Dale 2001; Dale et al. 2001). Finally, the faeder males grow breeding plumage typical of females, lacking ornamental feather growth and conspicuous plumage colors (Jukema and Piersma 2006) (Figure 1).

**Figure 1.**
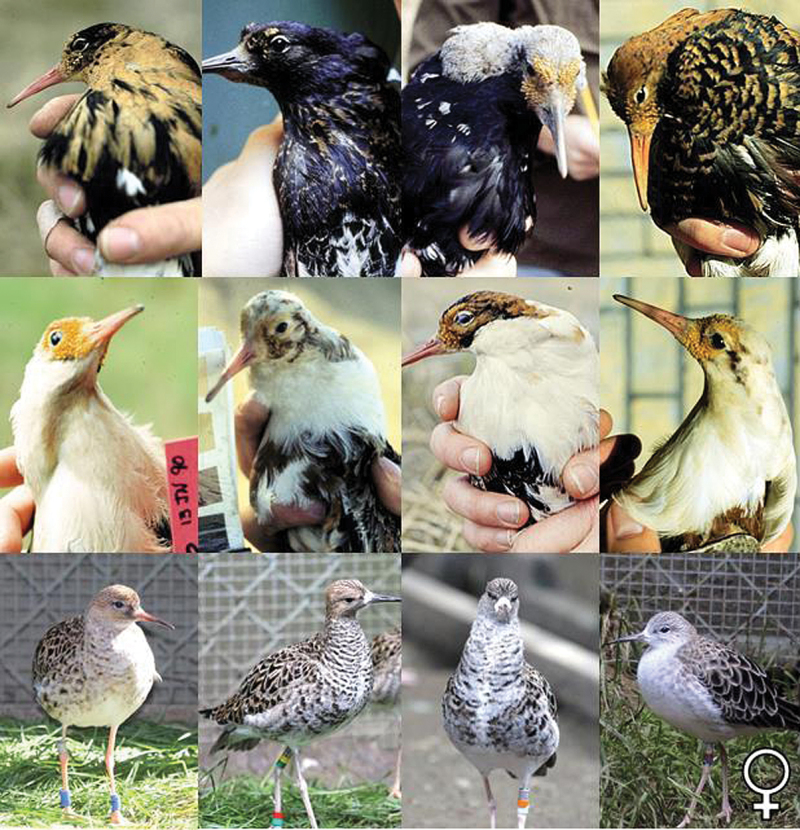
The wide range of melanic plumage coloration and patterns exhibited by male ruffs in their ornamental breeding plumage. Independents (top row) are predominantly dark in color, ranging from black, dark rust, light rust to ivory, with occasional patches of white. Satellites (middle row) are predominantly white in color and range from white, ivory to straw yellow, with secondary colors of light to medium rust, but lack solid black in the ruff or head tufts. Faeder males (bottom row) lack ornamental plumage and closely resemble the breeding plumage of ruff females (female far right) (Photos by D.B.L., C.K., and L.L.F.).

Sequence variation in the *melanocortin-1 receptor* (*MC1R*) gene explains major dark/light plumage color variation in several avian species as well as coat color variation in many mammals (Hubbard et al. 2010; Roulin and Ducrest 2013). Mutations that increase the activation of MC1R result in increased synthesis of eumelanin, producing shades of black or brown, whereas mutations reducing MC1R activation lead to increased synthesis of phaeomelanin producing shades of rust (Mundy 2005). For example, single base-pair mutations in the coding sequence of the *MC1R* gene perfectly associate with dark and light plumage types in bananaquit (*Coereba flaveola*), lesser snow goose (*Anser c. caerulescens*), Arctic skua (*Stercorarius parasiticus*) and chestnut-bellied monarch (*Monarcha castaneiventris*) (Uy et al. 2009; Theron et al. 2001; Mundy et al. 2004). In ruffs, we previously found that the presence/absence of male breeding plumage (the putative *Faeder* locus) (Lank et al. 2013) was associated with a microsatellite marker predicted to be in the close vicinity of *MC1R* (Farrell et al. 2013b), in addition to evidence of linkage disequilibrium between white ruff coloration and the *Satellite* locus (Farrell 2013). We therefore investigated whether coding sequence variation in the *MC1R* gene explains major dark/light breeding plumage color variation and/or the presence/absence of ornamental plumage in male ruffs.

## Methods

### Sample Population

Forty-four male ruffs with a diversity of breeding plumage phenotypes previously used in linkage mapping (Farrell et al. 2013a) were selected from a captive-bred population maintained by DBL at Simon Fraser University in Burnaby, British Columbia. The sample included 12 dark-plumed males, 12 light-plumed males, and 20 unornamented female-like faeder males (Table 1). The dark-plumed males are assumed to have higher levels of eumelanin within their dark feathers, whereas the light-plumed males are assumed to have phaeomelanin or lack pigmentation (pure white) (Van Rhijn et al. 2014). Unornamented individuals were included because of the apparent close linkage of the *Faeder* locus with a microsatellite marker located near the predicted location of *MC1R* (Farrell et al. 2013b).

**Table 1:** Behavioral morph, plumage type, plumage color of ruff, head tufts, and *MC1R* sequence data for 44 male ruff individuals included in this study

Ruff ID	Behavioral morph	Ornamental plumage type	Plumage color		SNP sites and consensus base		
			Ruff	*Head tufts*	*Thr93* A	*Val105* A	*His207Arg* A
302	Faeder	None	—	—	*	*	R
303	Faeder	None	—	—	R	*	R
304	Faeder	None	—	—	*	*	R
305	Faeder	None	—	—	R	*	R
307	Faeder	None	—	—	R	W	R
308	Faeder	None	—	—	R	*	R
310	Faeder	None	—	—	*	*	R
324	Faeder	None	—	—	*	*	R
336	Faeder	None	—	—	*	*	R
342	Faeder	None	—	—	R	*	R
353	Faeder	None	—	—	*	*	R
355	Faeder	None	—	—	*	*	R
672	Faeder	None	—	—	*	*	R
844	Faeder	None	—	—	*	*	*
845	Faeder	None	—	—	*	*	*
854	Faeder	None	—	—	R	*	*
855	Faeder	None	—	—	R	*	*
861	Faeder	None	—	—	R	*	*
3520	Faeder	None	—	—	*	*	*
5474	Faeder	None	—	—	G	*	*
211	Independent	Dark	I, Bl	Bl	*	W	R
241	Independent	Dark	Bl	Bl	*	*	R
261	Independent	Dark	Bl, DR	Bl	*	W	R
291	Independent	Dark	Bl	DR	R	*	*
294	Independent	Dark	Bl	DR	R	*	*
298	Independent	Dark	DR	Bl	R	W	R
306	Independent	Dark	LR, Bl	LR	R	*	*
314	Independent	Dark	LR, Bl	Br	R	W	R
325	Independent	Dark	LR,Bl	LR	*	W	R
328	Independent	Dark	Bl	LR	*	W	R
330	Independent	Dark	DR	Bl	*	*	*
363	Independent	Dark	MR, Bl	MR, Br	*	W	R
127	Satellite	Light	W	W	R	W	R
142	Satellite	Light	W, MR	MR	*	*	N/A
146	Satellite	Light	SY	SY	R	*	N/A
171	Satellite	Light	W	W	R	*	R
199	Satellite	Light	W	W	*	*	R
218	Satellite	Light	SY	SY	*	*	R
259	Satellite	Light	W	W	*	*	R
270	Satellite	Light	W	W	R	*	R
301	Satellite	Light	W	LR	R	*	R
312	Satellite	Light	W	DR	R	W	R
313	Satellite	Light	SY	SY	R	W	R
1241	Satellite	Light	W	W	*	W	R

* indicate agreement with the consensus sequence. All data were aligned and numbered with reference to the chicken *MC1R* sequence (Genbank AY220305). Plumage colors: W, white; I, ivory; Bl, black; Br, brown; DR, dark rust; MR, medium rust; LR, light rust; SY, straw yellow; *HT*, head tufts; heterozygotes denoted with standard IUPAC letter codes (R = A/G; W = A/T).

### MC1R Genotyping and Analysis

Genomic DNA was extracted from blood samples stored in absolute ethanol (50 μL of blood in 1.5mL of absolute ethanol) using an ammonium acetate precipitation method (Nicholls et al. 2000). A segment of the *MC1R* gene that encompassed sites previously associated with plumage polymorphism in birds was amplified using the conserved primers MSHR72 and MSHR9, with internal sequencing primers MSHR73 and MSHR74 (Mundy et al. 2004). Each 10-μL Polymerase Chain Reaction (PCR) contained approximately 10ng of genomic DNA, 1 μL of each primer (5 μM), 3 µL ultrapure H20 and 4 μL Qiagen Multiplex PCR Mix (Qiagen Inc.). PCR amplification was performed using a DNA Engine Tetrad 2 Thermal Cycler (MJ Research, BioRad, UK), with the following cycling parameters: 95 °C for 15min, followed by touchdown cycling 6× (95 °C for 30 s, 65 °C–60 °C for 20 s, 72 °C for 90 s), 30× (95 °C for 30 s, 60 °C for 20 s, and 72 °C for 90 s), and finally 72 °C for 10min. PCR products were treated with EXO-SAP (Illustra ExoStar) for 15min at 37 °C to remove unincorporated primers and dNTPs, followed by an inactivation step for 15min at 80 °C. Sequence reactions were ethanol/EDTA/sodium acetate precipitated (Sambrook et al. 1989). Purified template DNA was directly sequenced using Big Dye.v3.1 chemistry (PE Biosystems), according to the manufacturer’s protocol using an ABI3730 Genetic Analyzer (Applied Biosystems). Amplified fragments were sequenced in the forward and reverse complementary directions and a consensus sequence was created using a modified version of the Phred (Ewing et al. 1998; Ewing and Green 1998) and Phrap/Cross_match/Swat (Green 1996) software (PERL scripts provided by the NERC Biomolecular Facilty, UK). Sequences that did not form a consensus between their forward or reverse strands, but that were of good quality, were included in the study by alignment with those that did produce a consensus. Manual base calling and comparative analyses were performed in CODONCODE ALIGNER v 4.0 (http://www.codoncode.com/). Sequences were aligned in MEGA.v5.0 (Tamura et al. 2011) using ClustalW and sequences deposited in Genbank (Accession numbers LM993813-LM993852).

## Results

**Figure 2.**
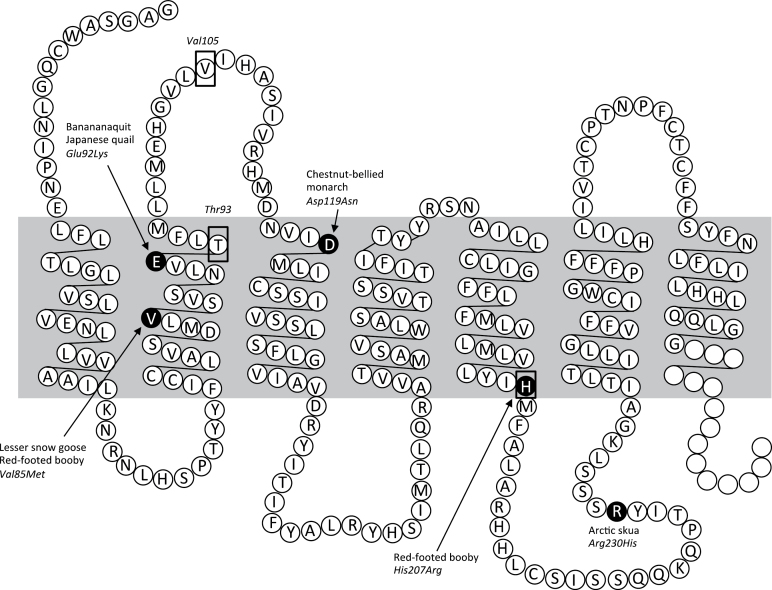
Partial amino acid sequence of the ruff (*Philomachus pugnax*) *melanocortin-1 receptor* gene (*MC1R*) with position of amino acids numbered after the chicken (*Gallus gallus*) (Kerje et al. 2003). The MC1R receptor is depicted in the cell membrane of a melanocyte (shaded in grey). Above is the extracellular region and below the intracellular region. Blank circles indicate partially missing data in *MC1R* sequence. Nonsynonymous SNPs associated with melanic plumage polymorphism in other bird species are shaded in black. Boxed amino acids indicate polymorphic SNPs detected in ruffs (Figure after Dobson et al. 2012).

A total of 821bp of the *MC1R* gene corresponding to positions 79–899 of the aligned chicken *MC1R* gene sequence (Kerje et al. 2003) was sequenced from 44 male ruff individuals. Three polymorphic SNPs were found: 2 synonymous substitutions (A/G: *Thr93*, A/T: *Val105*) and 1 nonsynonymous substitution (A/G: *His207Arg*); the latter polymorphism is shared with dark/light plumage differences in the red-footed booby (*Sula sula*) (Baião et al. 2007). At all the other functional sites known to be associated with dark and light melanic plumage variation in other species, all ruff *MC1R* sequences were monomorphic (Figure 2). None of the SNPs at the polymorphic ruff sites were associated with major dark/light plumage morphs or the presence/absence of breeding plumage (Table 1).

## Conclusion

More than 150 genes are known to affect animal coloration and pattern (Barsh 1996; Hoekstra 2006) and ruffs have the greatest naturally evolved intraspecific plumage diversity among birds (Lank and Dale 2001). Given that coding sequence variation in *MC1R* does not explain the major plumage color differences of male ruffs, we conclude that the control of plumage variation in ruffs is more complex than in other bird species with simple dark and light morphs. It is unlikely that the presence of dark or light coloration or the presence/absence of ornamental plumage in male ruffs is solely determined by amino acid variation within the *MC1R* locus. Because only a partial amino acid sequence of the coding region of the *MC1R* gene was sequenced, we cannot rule out the possibility that functional non-synonymous substitutions affecting plumage color may be present in the regions (approximately 9% based on other bird species) not sequenced. However, it seems more likely that regulatory polymorphisms account for the observed variation. The extensive individual variation suggests that ornamental plumage coloration is a polygenic trait, which might nonetheless involve *MC1R* along with other genes that affect the deposition of melanin (Dale et al. 2001; Roulin and Ducrest 2013; Van Rhijn et al. 2014). The potential functional significance, implied by the apparent proximity of the *Faeder* locus and *MC1R* (Farrell et al. 2013b), remains to be determined through more detailed mapping and analysis. Promoter regions of *MC1R* remain as candidates that might influence ornamental plumage type in ruffs. For example, local expression differences in *MC1R* and/or other pigmentation genes in the feather follicles of the neck ruff and head tufts could produce the observed hypervariability of the breeding plumage in this species.

## Funding

Natural Sciences and Engineering Research Council of Canada (NSERC to D.B.L.); UK Biotechnology and Biological Sciences Research Council grant (BB/J018937/1 to T.B.); NSERC PGS-D3 (L.L.F.); and Marie Curie Intra-European Fellowship (C.K.).
